# Does social prescribing address social determinants of health?

**DOI:** 10.3389/fpubh.2025.1531801

**Published:** 2025-03-06

**Authors:** M. Mofizul Islam

**Affiliations:** La Trobe University, Melbourne, VIC, Australia

**Keywords:** social prescribing, social determinants of health, inequality, structural determinants, social needs

## Introduction

Social prescribing allows healthcare professionals to refer and connect patients to various nonclinical services in their local areas (1). In recent years, social prescribing has gained traction in numerous countries, including Australia, Brazil, Canada, China, Ecuador, Japan, New Zealand, many European countries, Singapore, South Korea and the United States of America (2). The key reason for implementing social prescribing is that, in many instances, patients seek advice from healthcare professionals primarily for non-medical issues that are predominantly social (3, 4). Patients may present both non-medical and medical concerns at the same time, or they might perceive their problems as medical when they are actually social. Indeed, social and medical issues are often closely intertwined, and patients or even healthcare professionals may have difficulty distinguishing between them (5). As a result, if healthcare workers identify patients whose health is impacted by their social needs, they can refer them to social services, often in their local communities. Social services may include social networking, gardening, cooking, art, volunteering, befriending, support and advice on physical activities (6–8). These services may also help patients with concerns related to loneliness, mutual aid and parenting, job hunting, housing, financial hardship, acquiring new skills and legal issues.

The literature includes some arguments that social prescribing can help to address social determinants of health and related inequalities (9–12). Indeed, some scholars suggest that social prescribing offers a way to address health inequalities across various health settings as well as broader social determinants of health (13). Others argue further that social prescribing offers an opportunity to implement sustained structural changes in how clients navigate between professional sectors and integrate into their communities, thereby allowing them to address the social factors that influence their health (14). The government of the United Kingdom considers the reduction of health inequalities as a core principle of social prescribing and asserts that social prescribing will be effective at targeting the causes of health inequalities (15). In some settings in the United States, social prescribing is encompassed within the broader concept of social determinants of health care (16). However, there is disagreement about this, as some experts argue that while interventions that respond to the individuals under social prescribing are important, the claim that they address social determinants of health and related inequalities is problematic. Mackenzie et al. (17) view a need to dissociate the narratives of mitigating the effects of the social determinants of health from tackling the fundamental causes of health inequalities.

Based on existing literature, this paper discusses whether the social prescribing schemes address social determinants of health.

## Social determinants of health

It is important to start with the definition of social determinants of health. The World Health Organization defines it as the conditions in which people are born, grow, work, live and age, and the wider set of forces and systems shaping the conditions of daily life. These forces and systems include economic policies and systems, development agendas, social norms, social policies and political systems (18). Unfavorable conditions can result from a toxic combination of poor policies, ineffective programs, inequitable economic systems and inadequate governance. Inequality is a crucial part of it, although it is not always explicitly stated when discussing social determinants of health (19). Hence, a more accurate term would be social determinants of health and related inequalities (20).

**Figure 1:**
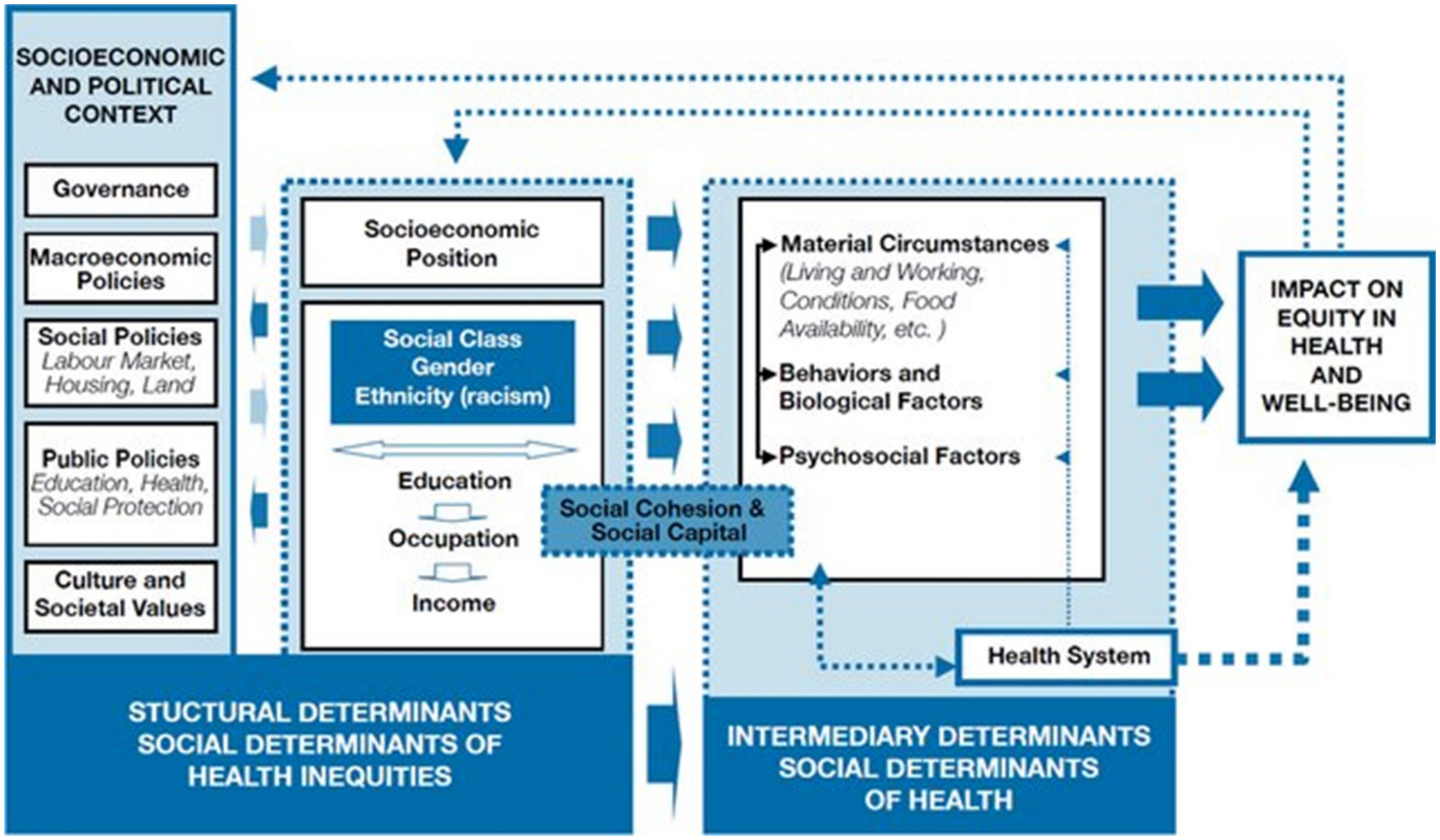
The World Health Organization (WHO)'s conceptual framework for social determinants of health; Source: Solar and Irwin (21). Reprinted with permission from the World Health Organization.

The framework for social determinants of health that was adopted by the WHO Commission on Social Determinants of Health (Figure 1) presents two interconnected sets of determinants: structural and intermediary, and it highlights the hierarchy of the determinants in relation to social and economic power (21). This framework also summarizes the pathways for the social determinants of health and related inequalities, from the socioeconomic and political contexts to the socioeconomic position (the structural determinants) and through to the impacts these have on intermediary determinants and ultimately on equity in health and wellbeing. Structural determinants are the causes of causes and are essential components of social determinants of health. Context, structural mechanisms and the resultant socioeconomic position, together are “structural determinants.” Some elements of the context, mainly societal and cultural values are embedded and work as a background. In relation to the social determinants of health, societal and cultural values refer to the value placed on health and the extent to which health is viewed as a collective socio-cultural concern within a society (21). Interventions addressing social determinants both at the structural and intermediary levels can have significant effects, potentially reducing inequalities within the entire system. The relationship between the three components, namely socioeconomic and political context, socioeconomic position and intermediary determinants (Figure 1) is similar to that between “structure” and “process” in Donabedian's Structure-Process-Outcome framework (22). A good structure increases the likelihood of good processes, the latter presupposes the former, and good processes increase the likelihood of good outcomes (22).

## Social prescribing does not address structural determinants

Providing targeted services through community initiatives under social prescribing, even on a large scale, is unlikely to have the same impact as making changes to structural determinants (23). In addition, the reliance of social prescribing on charities and community groups could lead to ineffective support in areas where there are few such organizations, especially when there are sudden fluctuations in community or third-sector resources that disproportionately affect areas with higher social deprivation (24). Therefore, even if social prescribing is effective for some patients, it may fail to help those who are most in need, and it could exacerbate existing inequalities unless it is delivered in a thoughtful way, with inequality being monitored and mitigated. The inverse care law posits that those who are most in need of care are least likely to receive it and vice versa (25). The patients who would benefit most from social prescribing usually include groups that are harder to reach and individuals who are less likely to engage, such as those who are socially isolated and struggling to establish supportive social relationships.

The connection between social prescribing and social determinants of health can be seen as a balance between agency and structure. The concept of social prescribing, like individual agency, suggests that individuals and communities can enhance their health and wellbeing by actively engaging in and accessing community resources (26). On the other hand, the concept of social determinants of health emphasizes the impacts of structural and systemic inequalities as the root causes of disparities in health outcomes (18). Social prescribing, as a form of individual or community-level endeavor, implies opportunities to organize and use community resources within the existing structure to enhance health and wellbeing at the individual level.

It is unrealistic to suggest that social prescribing should dismantle the ideology that places health as an individual responsibility regardless of the socio-economic context (27). Scott-Samuel and Smith (28) refer to the belief that inequities that result from broader structural issues can be eliminated through action at an individual/local level as fantasy paradigms. Indeed, addressing the social challenges that individuals face is a vastly different undertaking from addressing health determinants at a societal level, with vastly differing relevance for the inequitable distribution of health across society. The claim that social prescribing addresses social determinants of health inequities is more of a diversion from the actual changes that are required to reduce unequal health outcomes (23). Because of such unrealistic claims, then, some experts fear that social prescribing might become a distraction that allows policymakers to give the appearance of addressing social determinants of health and related inequalities without addressing upstream factors (12, 23, 24). To this end, Mackenzie et al. (17) call for a de-coupling of the public policy aspiration of reducing health inequalities from the operationalisation of social prescribing.

## Social prescribing offers services in downstream

There appears to be a conceptual ambiguity regarding the meaning of upstream factors in the context of social determinants of health. This ambiguity may explain some assertions such as that social prescribing moves care upstream to address social determinants of health through self-determination and supported referrals to community, volunteer and social services (2). Structural determinants within socioeconomic and political contexts are the top-level upstream factors. Offering social services to a group of people who have social needs and/or referring them to community social services under social prescribing schemes is unlikely to ensure favorable social determinants of health for all. In addition, there is a huge difference between the types of activities that social prescribing covers, such as volunteering, arts activities, group learning, gardening, making friendships, cooking, learning about healthy eating, sports activities, and the changes required to address social determinants of health and related inequalities (23, 29). While these social prescribing schemes can provide crucial assistance for patients facing toxic exposure to unfavorable social determinants of health, they cannot address upstream wealth and power inequities.

The concept of social prescribing recognizes that some individuals benefit from receiving social services downstream. However, certain policy documents suggest that social prescribing puts the responsibility for addressing the root causes of health and disease solely on the individual and at community levels. This perspective undermines the fact that social determinants are the fundamental causes of health issues that extend beyond the control of individuals (30). The social determinants of health have a population dimension that necessitates widespread change to ensure congenial circumstances in which people are born, grow, work, live and age. Although social prescribing could, in theory, be implemented in all communities, practical limitations may make this unfeasible. In addition, the scope and availability of social prescribing services differs depending on the locations. Relying solely on a grassroots approach in certain communities with varying service levels and coverage is not sufficient for addressing social determinants of health inequities on a national scale.

Social prescribing refers to a targeted approach to providing specific support services. However, many of these targeted strategies are inherently associated with the issue of labeling, which can deter some of the potential users from seeking help (31, 32). Indeed, the advantages of social prescribing may not be realized if those in need of social care are reluctant to use these services. Conversely, the concept of social determinants of health emphasizes addressing the health issues of an unhealthy society rather than focusing solely on unhealthy individuals within that society. If social determinants of health are addressed through upstream actions, the entire system should work, individuals' choices will matter little, and the benefits will reach all.

## Possible reasons for promoting social prescribing to address social determinants of health

Why, then, do some policymakers promote social prescribing as a way to address social determinants of health? The most likely explanation is that, while it may be politically convenient for governments to acknowledge health inequalities and the need for action within the framework of neoliberalism and a market-driven economy, they are unlikely to take genuine steps to tackle issues such as power imbalances, social status and class inequality. These structural issues are essential for reducing inequalities effectively (28). This inaction can, to some extent, be attributed to the potential unknowns that result from structural changes. I believe that most policymakers consider social determinants of health to be important, but that since they are often risk-averse, they prefer something like social prescribing to illuminate or promote and hence address social determinants of health. In addition, some policymakers may not have sufficient knowledge about social determinants of health and how to address them (20).

## Social prescribing is a natural approach to provide social support

However, this in no way means that social prescribing is valueless. Even for optimal scenarios where governments recognize the significance of social determinants of health and implement important policy changes, how can we determine whether the structural changes have affected social determinants of health and reduced inequality? Achieving zero inequality is unreasonable and, indeed, we do not know what level of inequality is acceptable. Within the best possible setting, there would still be some inequalities. Thus, interventions will be necessary at both the upstream and downstream levels. Social prescribing emerged as a natural reaction to meet some of the downstream needs, many of which were caused by unequal upstream socio-political structures. Indeed, some health and social care professionals have been practicing social prescribing for centuries, although this concept has gained prominence lately as its advocates have tried to semi-formalize the process. It is unlikely that social prescribing functioned in past centuries or even earlier only to reduce upstream inequality. Social prescribing was more likely to have occurred as a natural course to provide social support for those in need, recognizing what was commonly known, that people's health is determined by socioeconomic factors, and that people who have access to social supports within their communities are healthier (33).

## Conclusion

Muhl et al. (34) report that social prescribing mitigates the impacts of adverse social determinants of health inequities by addressing non-medical, health-related social needs. However, experts who are actively engaged in research into social determinants may find even this relatively mild assertion of mitigating the impacts of adverse social determinants of health problematic. Additionally, mitigating the adverse impacts of structural faults is one thing, and repairing the structure responsible for causing these adverse effects in the first instance is another. It is important to clarify that this paper does not intend to diminish the positive role that social prescribing plays in improving social wellbeing and health but rather to highlight concerns regarding the exaggerated claims about its effectiveness in addressing social determinants of health inequities. The scale of the services under social prescribing schemes is so selective and setting-specific that we should exercise caution when trying to claim that it addresses social determinants of health and related inequalities. Clearly, social prescribing brings support to a small and often select group of people at the individual level. However, it is like a tiny drop compared to what the social determinants of health advocates expect to see happening. It must be made crystal clear that social prescribing is a small endeavor downstream that may temporarily mitigate some of the social problems that result in adverse health outcomes.
